# Icotinib antagonizes ABCG2-mediated multidrug resistance, but not the pemetrexed resistance mediated by thymidylate synthase and ABCG2

**DOI:** 10.18632/oncotarget.2102

**Published:** 2014-06-13

**Authors:** De-Shen Wang, Atish Patel, Suneet Shukla, Yun-Kai Zhang, Yi-Jun Wang, Rishil J. Kathawala, Robert W. Robey, Li Zhang, Dong-Hua Yang, Tanaji T. Talele, Susan E. Bates, Suresh V. Ambudkar, Rui-Hua Xu, Zhe-Sheng Chen

**Affiliations:** ^1.^ Department of Medical Oncology, Sun Yat-sen University Cancer Center, State Key Laboratory of Oncology in South China, Collaborative Innovation Center for Cancer Medicine, China; ^2.^ Department of Pharmaceutical Sciences, College of Pharmacy and Health Sciences, St. John's University, Queens, New York, USA; ^3.^ Laboratory of Cell Biology, Center for Cancer Research, National Cancer Institute, NIH, Bethesda, Maryland, USA; ^4.^ Medical Oncology Branch, Center for Cancer Research, National Cancer Institute, NIH, Bethesda, Maryland, USA; ^5.^ Biosample Repository Facility, Fox Chase Cancer Center, Philadelphia, PA 19111, USA

**Keywords:** Icotinib, ABCG2, Reversal of drug resistance, thymidylate synthase, Lung cancer

## Abstract

ABCG2 is a potential biomarker causing multidrug resistance (MDR) in Non-Small Cell Lung Cancer (NSCLC). We conducted this study to investigate whether Icotinib, a small-molecule inhibitor of EGFR tyrosine kinase, could interact with ABCG2 transporter in NSCLC. Our results showed that Icotinib reversed ABCG2-mediated MDR by antagonizing the drug efflux function of ABCG2. Icotinib stimulated the ATPase activity in a concentration-dependent manner and inhibited the photolabeling of ABCG2 with [^125^I]-Iodoarylazidoprazosin, demonstrating that it interacts at the drug-binding pocket. Homology modeling predicted the binding conformation of Icotinib at Asn629 centroid-based grid of ABCG2. However, Icotinib at reversal concentration did not affect the expression levels of AKT and ABCG2. Furthermore, a combination of Icotinib and topotecan exhibited significant synergistic anticancer activity against NCI-H460/MX20 tumor xenografts. However, the inhibition of transport activity of ABCG2 was insufficient to overcome pemetrexed resistance in NCI-H460/MX20 cells, which was due to the co-upregulated thymidylate synthase (TS) and ABCG2 expression. This is the first report to show that the up-regulation of TS in ABCG2-overexpressing cell line NCI-H460/MX20 may play a role of resistance to pemetrexate. Our findings suggested different possible strategies of overcoming the resistance of topotecan and pemetrexed in the NSCLC patients.

## INTRODUCTION

Multidrug resistance (MDR) in cancer is a major impediment for successful chemotherapy [1]. Currently, 48 different ABC transporters have been identified in the human genome and classified into 7 subfamilies (A-G) based on similarities in sequence as well as structural organization [2]. ABCG2 transporter is a 72 kDa half transporter which was identified from a doxorubicin-selected MCF-7 human breast cancer cell line [3], human placenta [4], and a colon cancer cell line (S1-M1-80) [5]. Drugs transported by ABCG2 include a variety of anticancer agents such as mitoxantrone, topoisomerase I inhibitors, anthracyclines, indolocarbazole, flavopiridol, antifolates and fluorescent dyes like Hoechst 33342 [6].

Recently, ABCG2 has been recognized as a molecular marker for the side population (SP) cells [7]. As for human Non-Small Cell Lung Cancer (NSCLC) cell lines, the presence of a Hoechst dye 33342 extruding in SP cells, which accounted for 0.03 - 6.1% of the tumor cells [8], have shown elevated expression of ABCG2, increased tumorigenicity in mice, and resistance to various chemotherapeutic agents [9]. Moreover, Yoh K et al. [10] found that positive immunostaining for ABCG2 appears to be a predictor of shorter survival in patients with advanced NSCLC. Recently, a phase III study demonstrated that pemetrexed/cisplatin was a standard regimen for first-line treatment of advanced non-squamous NSCLC [11]. However, some studies reported that ABCG2 also confer pemetrexed resistance [12,13], which implied that blocking ABCG2-mediated active efflux function might substantially contribute to increased response and prolonged survival rates in patients with NSCLC [14].

Icotinib (BPI-2009H) is a specific small-molecule inhibitor of epidermal growth factor receptor (EGFR) tyrosine kinase, which has shown clinical anticancer activity in patients with advanced NSCLC [15]. However, there are still no biomarkers that reproducibly predicted the benefit of EGFR inhibitors in EGFR wild-type NSCLC patients, although they only produced a modest benefit in these patients [16]. In recent years, Gefitinib (ZD1839) and Erlotinib (OSI-774) have been shown to reverse ABCG2-mediated MDR [17,18]. It is conceivable that Icotinib might also inhibit the functions of ABC transporters by binding to the drug-binding domain. Therefore, we conducted this study to determine whether Icotinib could enhance the chemosensitivity of conventional anticancer drugs through interaction with ABCG2-mediated drug resistance in MDR NSCLC. We hypothesized that Icotinib might enhance the chemosensitivity of topotecan and pemetrexed in NSCLC.

## RESULTS

### Effect of Icotinib induced reversal MDR in various MDR cells

**Fig. 1:**
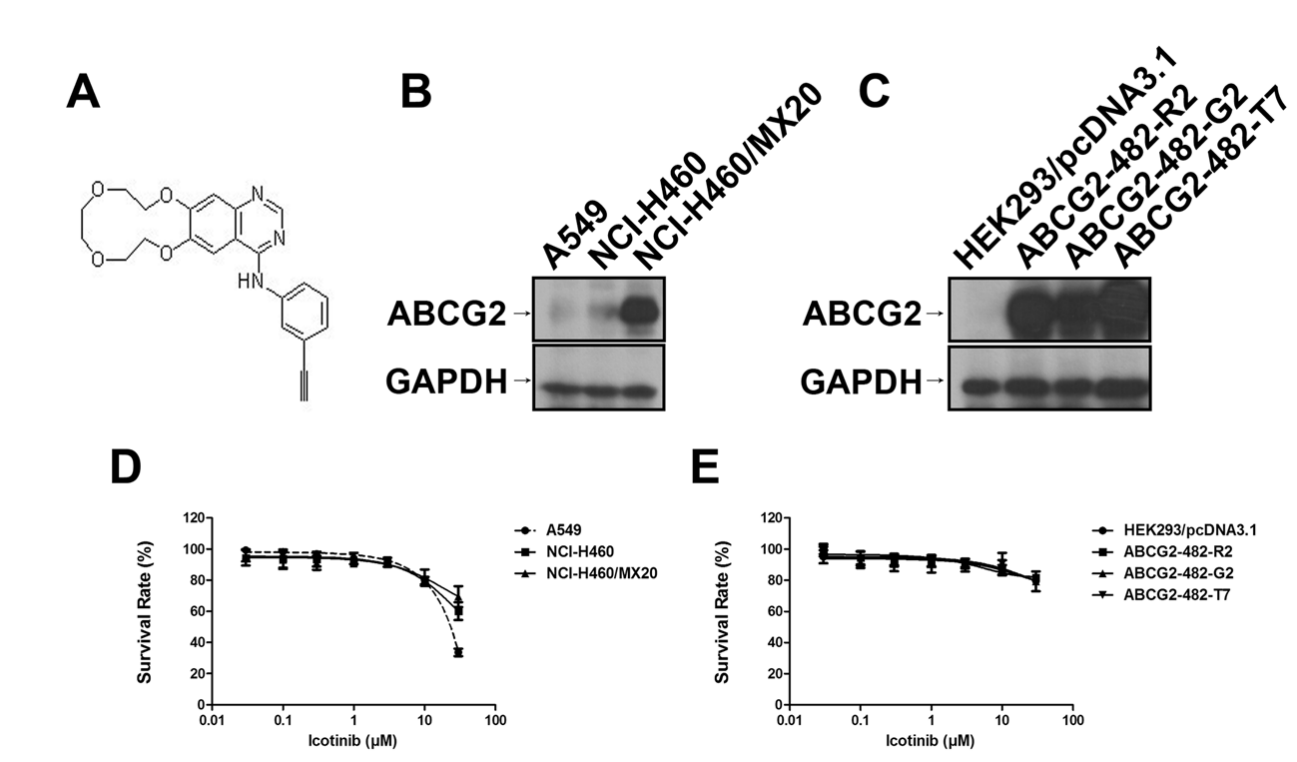
Chemical structure of Icotinib (A), Western blotting to detect ABCG2-expression in NCI-H460, A549 cells and in ABCG2-overexpressing NCI-H460/MX20 cell line (B) and transfected ABCG2-482-R2, ABCG2-482-G2, and ABCG2-482-T7 cell lines (C). Fig. 1 (D) and (E) represent the concentration-response curves of the cell lines treated with Icotinib alone.

Firstly, we detected the ABCG2 expressing levels in the cells used in this study. Low levels of ABCG2 were intrinsically expressed in lung cancer cell lines NCI-H460 and A549 (Fig. 1B). However, NCI-H460/MX20, ABCG2-482-R2, ABCG2-482-G2, and ABCG2-482-T7 cell lines showed much higher levels of ABCG2 expression (Fig. 1B and 1C), whereas the expression level of ABCG2 in the parental HEK293/pcDNA3.1 cell was undetectable (Fig. 1C). Secondly, we examined the reversal effect of Icotinib in ABCG2-mediated MDR cell lines (Table 1 and Table 2). In order to avoid the cytotoxicity of Icotinib alone in the reversal experiments, the concentrations of Icotinib we used were 1.0 and 5.0 μM, at which about 85% of cells were viable. As shown in Table 1, NCI-H460/MX20 cells possessed high resistance to the MX, SN-38 and topotecan that are substrates of ABCG2, compared with the NCI-H460 and A549 cells. Similarly, the ABCG2 transfected cell lines ABCG2-482-R2, ABCG2-482-G2 and ABCG2-482-T7 also possessed resistance to mitoxantrone (MX), SN-38 and topotecan compared with HEK293/pcDNA3.1 cells (Table 2). However, Icotinib at 5.0 μM significantly reversed the resistance of MX, SN-38 and topotecan in both drug selected derivative and transfected ABCG2-overexpressing cells, as well as the intrinsically ABCG2 expressed A549 and NCI-H460 cells, and its reversal efficacy was comparable to the specific ABCG2 inhibitor Fumitremorgin C (FTC) (5.0 μM). However, the reversal efficacy by Icotinib was not seen in HEK293/pcDNA3.1 cells. Meanwhile, there was no significant difference in the IC_50_ values for cisplatin, a non-substrate of ABCG2, with or without the combination of Icotinib in all tested cell lines (Table 1 and Table 2).

**Table 1:** Icotinib reverse the ABCG2-mediated drug resistance in drug selected resistant NSCLC cells

	IC_50_ ± SD^a^ (μM)					
Compounds	A549	(RF)^b^	NCI-H460	(RF)^b^	NCI-H460/MX20	(RF)^b^
Mitoxantrone (μM)	0.2853 ± 0.0214	1	0.0668 ± 0.0033	1.0	5.0837 ± 0.7542	76.0
+ Icotinib 1.0 μM	0.2269 ± 0.0193	0.8	0.0354 ± 0.0028*	0.5	1.9336 ± 0.1121*	28.9
+ Icotinib 5.0 μM	0.1695 ± 0.0102*	0.6	0.0314 ± 0.0022*	0.5	0.3559 ± 0.0249*	5.3
+ FTC 5.0 μM	0.1858 ± 0.0167*	0.7	0.0300 ± 0.0023*	0.4	0.3316 ± 0.0265*	5.0
SN-38 (μM)	0.6251 ± 0.0313	1.0	0.0707 ± 0.0035	1.0	8.1222 ± 1.006	114.9
+ Icotinib 1.0 μM	0.2482 ± 0.0199*	0.4	0.0202 ± 0.0016*	0.3	2.6770 ± 0.1553*	37.8
+ Icotinib 5.0 μM	0.1717 ± 0.0120*	0.3	0.0150 ± 0.0011*	0.2	0.3271 ± 0.0229*	4.6
+ FTC 5.0 μM	0.1752 ± 0.0131*	0.3	0.0166 ± 0.0012*	0.2	0.3126 ± 0.0250*	4.4
Topotecan (μM)	1.4551 ± 0.1237	1.0	0.0490 ± 0.0042	1.0	4.8721 ± 0.4141	99.4
+ Icotinib 1.0 μM	0.9458 ± 0.0851*	0.7	0.0226 ± 0.0020*	0.5	0.1261 ± 0.0113*	2.6
+ Icotinib 5.0 μM	0.9313 ± 0.0931*	0.6	0.0196 ± 0.0019*	0.4	0.0598 ± 0.0060*	1.2
+ FTC 5.0 μM	1.0186 ± 0.0866*	0.7	0.0194 ± 0.0016*	0.4	0.0639 ± 0.0054*	1.3
Cisplatin (μM)	2.7677 ± 0.2214	1.0	1.2692 ± 0.1015	1.0	1.3200 ± 0.1056	1.0
+ Icotinib 5.0 μM	2.9831 ± 0.1492	1.1	1.2727 ± 0.0636	1.0	1.2012 ± 0.0841	0.9
+ FTC 5.0 μM	3.2343 ± 0.2264	1.2	1.2017 ± 0.0841	0.9	1.2540 ± 0.0627	1.0

a IC_50_ values are represented the mean ± standard deviation (SD).

b Resistance fold (RF) was calculated by the IC_50_ values for different substrates, and cisplatin of NCI-H460/MX20 cells, divided by the IC_50_ values for the respective control without the reversing agents, or the resistant cell lines in the presence or absence of Icotinib or FTC, divided by the IC_50_ values for different substrates, and cisplatin of respective control without the reversing agents.

* . P < 0.05, versus the control group

In order to determine the reversal specificity by Icotinib, we examined the reversal effect in ABCB1- and ABCC10-mediated MDR cell lines. As shown in Supplementary Table S, Icotinib at 5.0 μM did not reverse ABCB1- and ABCC10-mediated drug resistance in HEK/ABCB1 and HEK/ABCC10 cell lines. Our results suggested that Icotinib probably reverse ABCG2-mediated drug resistance selectively.

### Icotinib increased intracellular accumulation of [^3^H]-MX and inhibited the efflux of [^3^H]-MX in cells over-expressing ABCG2

**Fig. 2:**
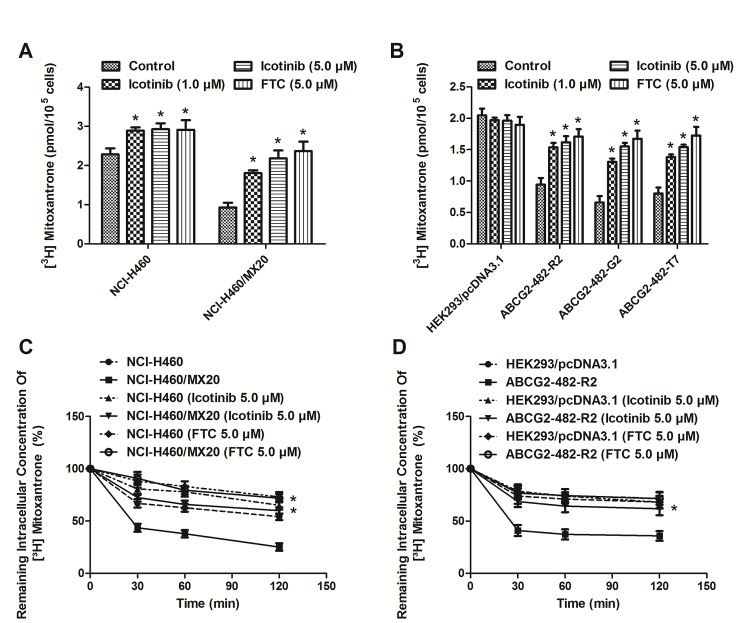
Effect of Icotinib on the accumulation and efflux of [^3^H]-MX in ABCG2-expressing cells Icotinib (at 1.0 and 5.0 μM) significantly increased intracellular accumulation of [^3^H]-MX in both the NCI-H460 and NCI-H460/MX20 (A) cells, as well as the transfected ABCG2-482-R2, ABCG2-482-G2, and ABCG2-482-T7 cells (B). The efflux activity of ABCG2 was significantly inhibited by 5.0 μM of Icotinib at 0, 30, 60, and 120 min of treatment in NCI-H460 and NCI-H460/MX20 (C), as well as the ABCG2-482-R2 (D) cells. All values are the mean ± SD of 3 assays. Columns, mean; bars, SD; *, *P* < 0.05, versus the respectively untreated controls.

As shown in Fig. 2 (A and B), the intracellular accumulation of [^3^H]-MX was significantly higher in NCI-H460 and HEK293/pcDNA3.1 cells than that in ABCG2 overexpressing NCI-H460/MX20, ABCG2-482-R2, ABCG2-482-G2, and ABCG2-482-T7 cells. Icotinib (1.0 and 5.0 μM) significantly increased intracellular accumulation of [^3^H]-MX in both the NCI-H460 and NCI-H460/MX20 cells, as well as the ABCG2-482-R2, ABCG2-482-G2, and ABCG2-482-T7 cells, and its effects were well comparable to that of FTC (5.0 μM). However, no change was seen in HEK293/pcDNA3.1 cells. In order to determine whether Icotinib could inhibit the drug efflux function of ABCG2, we examined the efflux of [^3^H]-MX in ABCG2-overexpressing cells. As shown in Fig 2 (C and D), the time course of release of [^3^H]-MX in ABCG2-overexpressing cells ABCG2-482-R2 and NCI-H460/MX20 was significantly higher than that of the HEK293/pcDNA3.1 and NCI-H460 cells, respectively. However, Icotinib at 5.0 μM significantly inhibited the efflux of [^3^H]-MX at different time points (30, 60, and 120 min) of treatment in NCI-H460/MX20 and ABCG2-482-R2 cells, as well as the NCI-H46 cells, but showed no apparent effect in the treatment of parental HEK293/pcDNA3.1 cells.

### Effect of Icotinib on the protein expression of AKT, pAKT, ABCG2 and the cellular localization of ABCG2

**Fig. 3:**
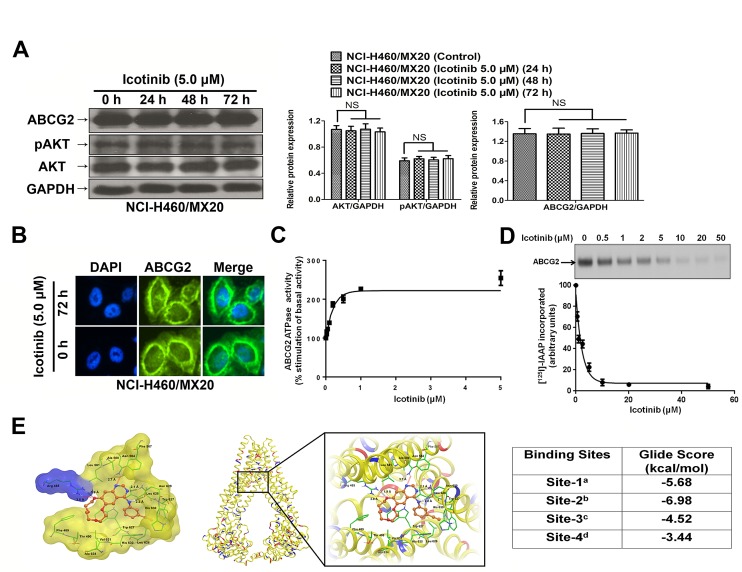
The effect of Icotinib on the expression levels of pAKT, total AKT, ABCG2, the subcellular localization of ABCG2, ATPase activity, the photoaffinity labeling with [^125^I]-IAAP, and its docking in the homology model of ABCG2 A. Effect of Icotinib at 5.0 μM on the expression level of pAKT, total AKT, and ABCG2 in NCI-H460/MX20 cell line. The protein levels of AKT, pAKT and ABCG2 were normalized to those of GAPDH in the NCI-H460/MX20 cell lines. Values are the mean ± SD of 3 assays. Columns, mean; bars, SD; NS, not significant. B. Effect of Icotinib treatment on the subcellular localization of ABCG2 in NCI-H460/MX20 cell. ABCG2 staining is shown in green. DAPI (blue) counterstains the nuclei. C. Effect of Icotinib on the ATPase activity of ABCG2: The BeFx-sensitive specific ATPase activity of ABCG2 was determined in the presence of 0-5 μM of Icotinib as described in supplemental methods. The activity in the absence of Icotinib (basal activity) was considered to be 100%, and % -fold stimulation ± S.D. (Y-axis) was plotted as a function of indicated concentrations of Icotinib (X-axis). D. Effect of Icotinib on the photolabeling of ABCG2 with [^125^I]-IAAP: Crude membranes from ABCG2 expressing MCF7-FLV1000 cells were photo-crosslinked with [^125^I]-IAAP in the presence and absence of 0-50 μM of Icotinib as described in supplemental methods. [^125^I]-IAAP incorporated in ABCG2 band was quantified using ImageQuant software and plotted as % [^125^I]-IAAP incorporated ± S.D. (Y-axis) as a function of varying concentration of Icotinib (X-axis). The upper panel shows a representative autoradiogram from three independent experiments and the arrow represents the ABCG2 band photo-crosslinked with [^125^I]-IAAP. E. XP Glide predicted binding model of Icotinib with homology modeled ABCG2. The docked conformation of Icotinib as ball and stick model is shown within the large drug-binding cavity of ABCG2. Important amino acids are depicted as sticks with the atoms colored as carbon-green, hydrogen-white, nitrogen-blue, oxygen-red, whereas Icotinib is shown with the same color scheme as above except carbon atoms are represented in orange. Dotted black line indicates hydrogen bonding interactions, whereas dotted red line indicates electrostatic interactions. Left: ABCG2 is represented as Macromodel surface based on residue charge (hydrophobic-yellow, basic-blue). Middle: ABCG2 is represented as protein ribbons based on residue charge (hydrophobic-yellow, basic-blue, acidic-red). Right: Binding energies of Icotinib within each of the predicted binding sites of ABCG2. ^a^Site grid generated using Arg482; ^b^Site grid generated using Asn629; ^c^Site grid generated using Arg383; ^d^Site grid generated using Leu241 and Gly83.

The expression levels of ABCG2 were examined to evaluate if Icotinib could alter the expression levels of ABCG2 and its related prosurvival kinase AKT (Fig. 3A). Our results found that the protein expression levels of ABCG2 and pAKT were not significantly different from that in the ABCG2 overexpressing NCI-H460/MX20 cell line, when treated with Icotinib (5.0 μM) at 24, 48 and 72 h compared with the untreated cells. Furthermore, the immunofluorescence assay showed that, with up to 72 h treatment of Icotinib at 5.0 μM, Icotinib did not significantly modulate the re-localization of ABCG2 from cell membrane to internal compartments in the NCI-H460/MX20 cells (Fig. 3B).

**Table 2:** The reversal efficacy of Icotinib in ABCG2-mediated drug resistance in ABCG2-transfected cell lines

	IC_50_ ± SD^a^ (μM)							
Compounds	HEK293/pcDNA3.1	(RF)^b^	ABCG2-482-R2	(RF)^b^	ABCG2-482-G2	(RF)^b^	ABCG2-482-T7	(RF)^b^
Mitoxantrone (μM)	0.0557 ± 0.0028	1.0	0.5754 ± 0.0460	10.3	1.7045 ± 0.1364	30.6	1.1045 ± 0.0884	19.8
+ Icotinib 1.0 μM	0.0533 ± 0.0037	1.0	0.3438 ± 0.0309*	6.2	0.7934 ± 0.0714*	14.3	0.5524 ± 0.0497*	9.9
+ Icotinib 5.0 μM	0.0503 ± 0.0040	0.9	0.0537 ± 0.0027*	1.0	0.0701 ± 0.0035*	1.3	0.0687 ± 0.0034*	1.2
+ FTC 5.0 μM	0.0491 ± 0.0022	0.9	0.0564 ± 0.0023*	1.0	0.0645 ± 0.0026*	1.2	0.0632 ± 0.0025*	1.1
								
SN-38 (μM)	0.0070 ± 0.00035	1.0	0.1885 ± 0.0151	27.1	0.2494 ± 0.0200	35.8	0.2095 ± 0.0168	30.1
+ Icotinib 1.0 μM	0.0067 ± 0.0005	1.0	0.0612 ± 0.0055*	8.8	0.0811 ± 0.0073*	11.7	0.0750 ± 0.0068*	10.8
+ Icotinib 5.0 μM	0.0063 ± 0.0005	0.9	0.0087 ± 0.0004*	1.2	0.0080 ± 0.0004*	1.2	0.0077 ± 0.0005*	1.1
+ FTC 5.0 μM	0.0061 ± 0.0003	0.9	0.0075 ± 0.0003*	1.1	0.0078 ± 0.0003*	1.1	0.0078 ± 0.0003*	1.1
								
Cisplatin (μM)	1.6700 ± 0.0835	1.0	1.7045 ± 0.1364	1.0	1.6045 ± 0.1284	1.0	1.6704 ± 0.1336	1.0
+ Icotinib 5.0 μM	1.5980 ± 0.1119	1.0	1.6662 ± 0.1500	1.0	1.5524 ± 0.1397	0.9	1.5500 ± 0.0775	0.9
+ FTC 5.0 μM	1.5098 ± 0.1208	0.9	1.5801 ± 0.0790	0.9	1.6873 ± 0.0844	1.0	1.5400 ± 0.1001	0.9

a. IC_50_ values are represented the mean ± standard deviation (SD).

b. Resistance fold (RF) was calculated by the IC_50_ values for different substrates, and cisplatin of resistant cell lines, divided by the IC_50_ values for the respective control without the reversing agents, or the resistant cell lines in the presence or absence of Icotinib or FTC, divided by the IC50 values for different substrates, and cisplatin of respective control without the reversing agents.

* . P < 0.05, versus the control group.

### Icotinib interacts at the drug-binding pocket of ABCG2

The above data indicated that Icotinib might inhibit the ABCG2-mediated efflux of the cytotoxic drugs by binding to the drug-binding pocket of the ABCG2 transporter. To further confirm Icotinib's interaction with ABCG2, its effect was evaluated on the photo-crosslinking of ABCG2 with [^125^I]-Iodoarylazidoprazosin (IAAP) (an ABCG2 substrate) and ATPase activity of this transporter. As shown in Fig. 3C and, Icotinib inhibited the photo-crosslinking of ABCG2 with [^125^I]-IAAP in a concentration-dependent manner with an IC_50_ value of 1.61 μM and stimulated the ATPase activity of this transporter in a concentration-dependent manner with a maximal stimulation of 2.54-fold. The concentration of Icotinib required for 50% of maximal stimulation was 0.14 μM. These results demonstrated that similar to other TKIs, Icotinib also interacts at the substrate-binding pocket of ABCG2.

### Docking of Icotinib in the homology model of ABCG2

The highest score docked model of Icotinib at Asn629 centroid grid of ABCG2 showed the importance of hydrophobic and hydrogen-bonding interactions (Fig. 3E). The 3-ethinylphenyl ring was stabilized by nearby residues Leu626, Trp627 and His630 through hydrophobic interactions. The backbone of Asn629 formed two hydrogen bonding interactions with the -NH- linker (-NH···OC-Asn629, 2.2 Å) and the N_3_ atom of the quinazoline ring (N_3_···H_2_N-Asn629, 2.1 Å). Quinazoline ring was stabilized by the side chains of Phe507, Ala580, Leu581, Asn584, Leu626 and Try627. The N_1_ atom of quinazoline ring formed an electrostatic interaction with the side chain amino group of Asn584 (N_1_···H_2_N-Asn584, 3.7 Å). The 2, 3, 5, 6, 8, 9-hexahydro-1, 4, 7, 10-tetraoxacyclododecanol ring was stabilized by Thr490, Phe489, Val631 and Ala634. The O_1_ atom of this macrocycle interacted with the guanidine group of Arg465 by both hydrogen-bonding interaction (O_1_···H_2_N-Arg465, 1.9 Å) and electrostatic interaction (O_1_···H_2_N-Arg465, 3.0 Å).

### Inhibition of ABCG2 function by Icotinib did not sensitize NCI-H460/MX20 cells to pemetrexed

**Fig. 4:**
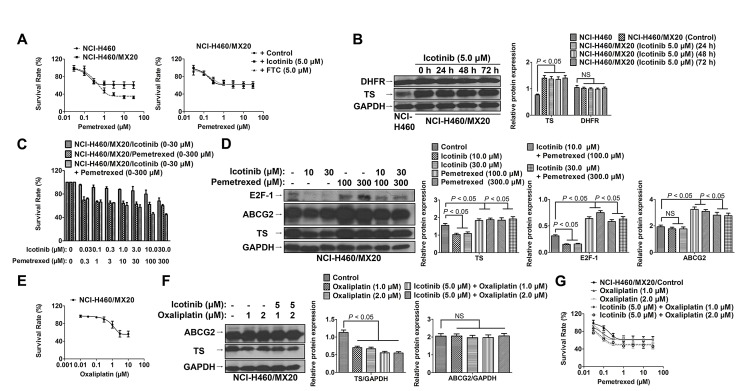
Icotinib did not sensitize NCI-H460/MX20 cells to pemetrexed A. The cytotoxicity effect of pemetrexed in the NCI-H460 and NCI-H460/MX20 cell lines. Icotinib (at 5.0 μM) did not significantly reverse pemetrexed resistance in the NCI-H460/MX20 cells. B. Western blot analysis was conducted to examine the expression levels of TS and DHFR between the NCI-H460 and NCI-H460/MX20 cell lines. NCI-H460/MX20 cells were treated with Icotinib at 5.0 μM up to 72 h. The protein levels of TS and DHFR were normalized to those of GAPDH in the NCI-H460 and NCI-H460/MX20 cell lines. Values are the mean ± SD of 3 assays. Columns, mean; bars, SD; NS, not significant. C. The cytotoxicity effect of combined serial concentrations of Icotinib (0 - 30 μM) with pemetrexed (0 - 300 μM) in NCI-H460/MX20 cells. Icotinib (30 μM) plus pemetrexed (300 μM) did not induce more synergistic effect than a combination of Icotinib (10 μM) plus pemetrexed (100 μM). D. Western blot analysis to examine the expression levels of TS, ABCG2 and E2F-1 when treated with either Icotinib alone (at 10 μM and 30 μM), pemetrexed alone (at 100 μM and 300 μM), or their combination. The protein levels of TS, ABCG2 and E2F-1 were normalized to those of GAPDH. Values are the mean ± SD of 3 assays. Columns, mean; bars, SD. E. The cytotoxicity effect of oxaliplatin in the NCI-H460/MX20 cell line. F. Western blot analysis was conducted to examine the expression levels of TS and ABCG2 when treated with either oxaliplatin alone (1.0 or 2.0 μM), or Icotinib (5.0 μM) and oxaliplatin (1.0 or 2.0 μM) combination. The protein expression levels of TS and ABCG2 were normalized to those of GAPDH. Values are the mean ± SD of 3 assays. Columns, mean; bars, SD. G. The cytotoxicity effect of pemetrexed when combined with either oxaliplatin alone (1.0 or 2.0 μM), or Icotinib (5.0 μM) and oxaliplatin (1.0 or 2.0 μM) combination. Oxaliplatin alone (1.0 or 2.0 μM) could not significantly reverse pemetrexed resistance. However, the combination of Icotinib (5.0 μM) with oxaliplation (1.0 or 2.0 μM) could significantly enhance the chemosensitivity of pemetrexed in the NCI-H460/MX20 cells.

As shown in Fig. 4A, NCI-H460/MX20 showed high resistance to the pemetrexed compared to that of the parental cells NCI-H460. However, Icotinib at 5 μM did not significantly reverse pemetrexed resistance in the NCI-H460/MX20 cells. Interestingly, the expression of thymidylate synthase (TS) was significant higher in the NCI-H460/MX20 cell line than in the NCI-H460 cells. Moreover, the TS expression was not significantly altered after treatment with Icotinib (5 μM) up to 72 h. However, the expression of dihydrofolate reductase (DHFR) was not significantly different between these two cell lines (Fig. 4B). Furthermore, when serial concentrations of Icotinib (0~30 μM) were combined with those of pemetrexed (0~300 μM), the more synergistic cytotoxicity of Icotinib and pemetrexed combination was not detected when compared Icotinib (30 μM) plus pemetrexed (300 μM) with Icotinib (10 μM) plus pemetrexed (100 μM) (Fig. 4C). More interestingly, Icotinib alone (at 10 and 30 μM) could significantly reduce TS expression, possibly through E2F-1 reduction. However, pemetrexed alone (at 100 and 300 μM) could significantly induce TS and ABCG2 expression. Nevertheless, the expression of TS was not additionally reduced by Icotinib and pemetrexed combination (Fig. 4D). Furthermore, previous study had shown that oxaliplatin could down-regulate the expression of TS [19]. In our study, oxaliplatin at the non-toxic concentration (1.0 and 2.0 μM) (Fig. 4E), could inhibit the TS expression in the NCI-H460/MX20 cells (Fig. 4F). The combination of Icotinib (5.0 μM) with oxaliplation (1.0 or 2.0 μM) could significantly enhance the chemosensitivity of pemetrexed in the NCI-H460/MX20 cells (Fig. 4G)

### Therapeutic effect of Icotinib combined with pemetrexed or topotecan *in vivo* NCI-H460/MX20 tumor xenografts

**Fig. 5:**
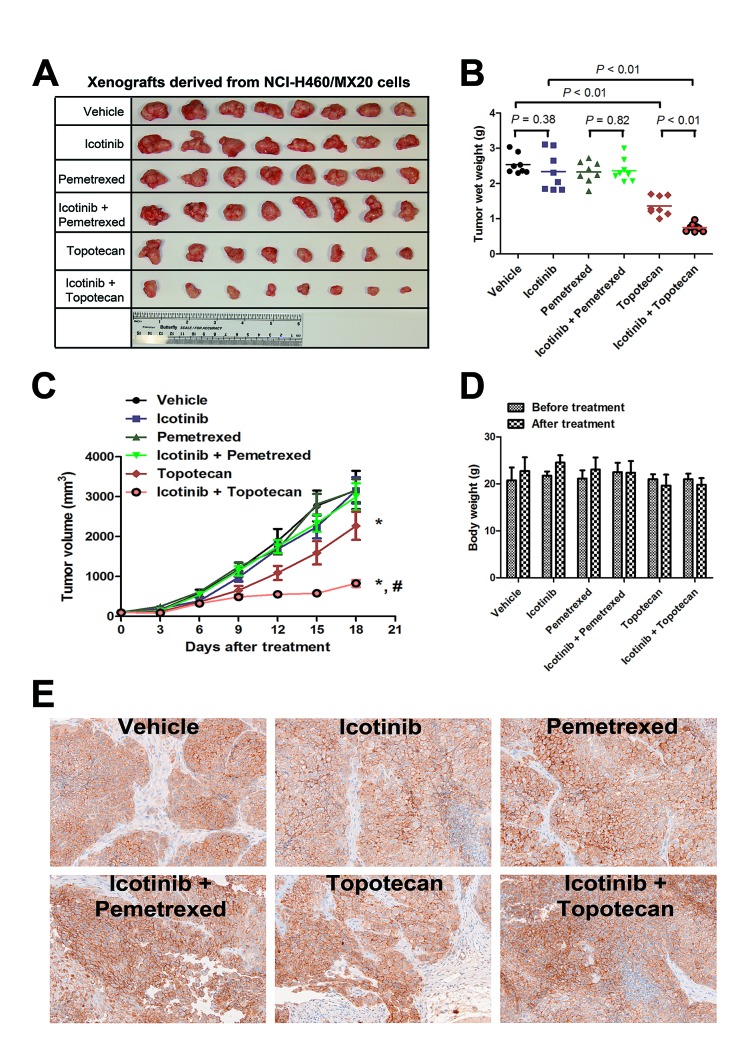
Therapeutic effect of Icotinib combined with pemetrexed or topotecan in a xenograft model of NCI-H460/MX20 cells in athymic nude mice The doses of each group were as follows: vehicle (q3d × 6); Icotinib (60 mg/kg, p.o., q3d × 6); pemetrexed (100 mg/kg, i.p., q3d × 6); Icotinib (60 mg/kg, p.o., q3d × 6, given 2 h before giving pemetrexed) + pemetrexed (100 mg/kg, i.p., q3d × 6); topotecan (3.0 mg/kg, i.p., q3d × 6); and Icotinib (60 mg/kg, p.o., q3d × 6, given 2 h before giving topotecan) + topotecan (3.0 mg/kg, i.p., q3d × 6). A. The tumors were excised and weighed, and the representative pictures of each group were taken after 18 days of treatment. B. Tumor weights (n = 8) of 6 groups are represented as each dot. Solid lines indicate the mean of each group. *P* values were calculated using the Student's t-test. C. The tumor volumes were measured every 3 days, and the tumor growth curve was created for each group (n = 8). Dots, mean tumor volume; bars, SD; *, *P* < 0.01 versus the vehicle group; #, *P* < 0.01 versus topotecan alone group. D. The body weights of mice were measured every 3 days, and changes in the mean body weight before and after treatment are shown in the bar graph. E. Immunohistochemistry analysis of ABCG2 expression in the tumor tissues from 6 groups after 18 days of treatment. ABCG2 specifically expressed in the cell membrane and the expression of ABCG2 was not significantly different among 6 treatment groups.

As shown in Fig. 5A and, treatment with topotecan as a single agent exhibited a significant suppression of tumor growth (*P* < 0.01). The inhibition rate of topotecan alone was 46.3%. The ratio of growth inhibition *P* Treatment with Icotinib alone also did not significantly decrease the tumor weight (*P* = 0.38). Strikingly, a combination of Icotinib and topotecan showed a dramatic synergistic anticancer effect when compared with either treatment of topotecan or Icotinib alone. The Icotinib and topotecan combination reduced the averaged tumor size by 70.5% (*P* < 0.01). However, the synergistic anticancer effect was not detected when the combination of Icotinib and pemetrexed compared with the control group (*P* = 0.28). Moreover, the *in vivo* therapeutic effect of Icotinib combined with pemetrexed or topotecan was further confirmed by the flat tumor growth curve (Fig. 5C). The Icotinib and topotecan combination caused a slight decrease in body weight, but this effect was not statistically significant (Fig. 5D). Moreover, Immunohistochemistry (IHC) analysis of the excised tumors showed that the expression of ABCG2 was not significantly different among 6 treatment groups (Fig. 5E).

## DISCUSSION

In the present study, we showed for the first time that Icotinib potentiated the chemosensitivity of established ABCG2 substrates in ABCG2-overexpressing cells. Icotinib treatment significantly increased the intracellular accumulation of [^3^H]-MX in both the NCI-H460 and NCI-H460/MX20 cell lines. Moreover, previous studies have found that the Arg482- to G482- or Thr482-ABCG2 mutation altered substrate specificity [20,21]. Our results found that like FTC, Icotinib significantly enhanced the chemosensitivity of ABCG2 substrates in both the cells with wild-type Arg482 and mutant-type G482 or Thr482 of ABCG2. Furthermore, Icotinib could not reverse ABCB1-, and ABCC10-mediated drug resistance, indicating that the reversal effect of Icotinib might attribute to its specific effect on the overexpression of ABCG2.

In human tumors, EGFR plays a crucial role in the biological behavior of NSCLC [22], and the overexpression of the EGFR is associated with the response to anti-EGFR agents [23]. Pick, A et al. [24] found that EGFR might exert a post-transcriptional enhancing effect on ABCG2 expression via the PI3K/AKT signaling pathway, and EGFR inhibitors might influence ABCG2 expression in EGFR-positive MDCK ABCG2 cells. Moreover, Goler-Baron, V et al. [25] reported that PI3K-AKT signaling pathway was a key regulator of subcellular localization of ABCG2 and functional MDR. Inhibition of AKT signaling pathway might result in relocation of ABCG2 from the cell membrane to the intracellular compartment [25,26]. Icotinib is an inhibitor of EGFR, which might indirectly modulate the phosphorylation of AKT via EGFR and its downstream signal pathway, and can further influence the ABCG2 expression thereby reversing ABCG2-mediated MDR. However, we found that Icotinib neither significantly alter the protein expression levels of pAKT, ABCG2 nor stimulate translocation of ABCG2, suggesting that the reversal of ABCG2-mediated MDR by Icotinib might be through inhibition of its transport activity, rather than modulation of the subcellular protein expression or translocation.

Tyrosine kinase inhibitors are known to interact at the substrate-binding pocket of ABC transporters [27]. The data from photo-crosslinking of ABCG2 with [^125^I]-IAAP and ATPase activity of ABCG2 demonstrate that Icotinib, similarly to other TKIs, interacts at the substrate-binding pocket of this transporter. The concentration of Icotinib required for 50% stimulation of maximal ABCG2 ATPase activity was 0.14 μM, suggesting that it has a relatively higher affinity for interaction at the substrate-binding pocket of this transporter compared to other TKIs [27]. Furthermore, docking simulations suggested several strong interactions between Icotinib and ABCG2 homology model, which was consistent with our experimental data. Predicted best-docked model at Asn629 grid may explain its insensitivity to Arg482 mutations. These strong contacts may be induced by previously illustrated essential pharmacophoric features for ABCG2 binding [28].

Recently, Yoh K et al. [10] demonstrated that overexpression of ABCG2 appeared to be a predictor of shorter survival in patients with advanced NSCLC. Currently, pemetrexed/cisplatin is a standard regimen for first-line treatment of advanced non-squamous NSCLC [11]. However, some studies report that pemetrexed is a substrate of ABCG2 [12,13], which implies that blocking ABCG2-mediated active efflux function might substantially contribute to increased response rates and prolonged survival in patients with NSCLC [14]. In our study, we found that the ABCG2-overexpressing NCI-H460/MX20 cells showed high resistance to the pemetrexed compared to the parental cell NCI-H460. However, Icotinib at 5.0 μM did not significantly reverse pemetrexed resistance in the NCI-H460/MX20 cells. Interestingly, we found that the TS expression, which was another major factor that mediated pemetrexed resistance [29], was significant higher in the NCI-H460/MX20 cells than in the NCI-H460 cells, and that in both these cell lines, the expression levels of TS were consistent with the expression of ABCG2. To the best of our knowledge, this is the first report to show that the up-regulation of TS in the MX-selected derivative ABCG2-overexpressing cell line NCI-H460/MX20. In the previous study, Salnikov et al. [30] found a significant association between the expression of TS and CD133, which represents a marker of tumor-initiating cells in NSCLC, and was co-expression of ABCG2 in subpopulation of NSCLC patients [31]. However, this co-upregulation phenomenon needs to be confirmed in other ABCG2-overexpressing cell lines, and the mechanism that mediates both the ABCG2 and TS up-regulation still needs to be examined in our further studies.

Until now, no predictive biomarkers to the combination of EGFR-TKIs with chemotherapy have been identified. In our study, we determined that ABCG2 might be a potential biomarker for the clinical outcome to a combination of Icotinib with ABCG2 substrates in patients with NSCLC. To determine whether the reversal efficacy of Icotinib *in vitro* could be extended to an *in vivo* xenografts model, we investigated the potentiation of Icotinib on the antitumor activity of topotecan or pemetrexed in NCI-H460/MX20 tumor xenograft model. Our results found that the combination of Icotinib with topotecan markedly enhanced antitumor activity of topotecan in the ABCG2-overexpressing NCI-H460/MX20 tumor xenografts model when compared to either the Icotinib or topotecan treatment alone. However, the synergistic antitumor activity was not observed in treatment with Icotinib and pemetrexed, which might be due to the inefficiency of Icotinib in overcoming the additional up-regulation of TS in the NCI-H460/MX20 tumor xenografts. In the previous study, Giovannetti et al. [32] found that Erlotinib and pemetrexed showed a strong synergism in NSCLC cells, possibly via the combination decreased TS expression and E2F-1. However, in our study, we found that Icotinib alone could significantly reduce TS expression, possibly through E2F-1 reduction. Pemetrexed alone could significantly increase the TS and ABCG2 expression. Nevertheless, the TS and ABCG2 expression were not reduced by Icotinib and pemetrexed combination, which finally led to the pemetrexed resistance in the NCI-H460/MX20 cells and xenografts. Furthermore, our results showed that the TS up-regulation could be overcome by oxaliplatin, and that the combination of oxaliplatin with Icotinib could significantly reverse pemetrexed resistance in the NCI-H460/MX20 cells. Our results suggested the inhibition of both the TS and ABCG2 expressions might contribute to the ability to overcome the resistance of pemetrexed in the TS- and ABCG2-overexpressing NSCLC patients.

In conclusion, the present study showed that Icotinib significantly reversed ABCG2-mediated MDR by directly inhibiting the drug transport activity of ABCG2. Combination of Icotinib and topotecan exhibited significant synergistic anticancer activity against the ABCG2-overexpressing NCI-H460/MX20 cells both *in vitro* and *in vivo*. Antagonizing the activity of ABCG2 was insufficient to overcoming pemetrexed resistance in NCI-H460/MX20 cells and xenografts, probably due to the co-upregulation of TS and ABCG2. Our study identified a promising therapeutic strategy in overcoming ABCG2-mediated drug resistance in NSCLC. However, the mechanism that up-regulated both the TS and ABCG2 in the MX-selected derivative ABCG2-overexpressing NSCLC cell line NCI-H460/MX20 needs to explored in further studies.

## METHODS

### Materials

Icotinib (Fig. 1A) was a product of Selleck Chem Inc (Houston,TX, USA). For the animal study, Icotinib hydrochloride tablets were a gift from Zhejiang Beta Pharma, Inc, China. MX, SN-38, topotecan, pemetrexed, vincristine, cisplatin, oxaliplatin, verapamil, dimethyl sulfoxide (DMSO) and 3-(4, 5-dimethylthiazole-2-yl)-2, 5-biphenyl tetrazolium bromide (MTT) and other chemicals were purchased from Sigma Chemical Co (St. Louis, MO, USA). [^125^I]-IAAP (2,200 Ci/mmol) was purchased from PerkinElmer Life and Analytical Sciences (Waltham, MA). Cepharanthine was provided by Kakenshoyaku Co. (Tokyo, Japan). FTC was provided by Dr. Susan E. Bates from NIH (Bethesda, Maryland, USA).

### Cell lines and cell culture

HEK293/pcDNA3.1, wild-type ABCG2-482-R2, mutant ABCG2-482-T7 and mutant ABCG2-482-G2 cells were established by transfecting HEK293 cell with either the empty pcDNA3.1 vector or pcDNA3.1 vector containing a full-length ABCG2, with coding arginine (R), threonine (T), or glycine (G) at amino acid position 482, respectively after selection with G418 and maintenance in the medium with 2 mg/ml of G418 [21]. HEK/ABCB1 and HEK/ABCC10 cell lines were generated by selection with G418 (2 mg/ml) after transfecting HEK293 cell with ABCB1 vector or ABCC10 vector, respectively [33]. The human lung cancer cell line NCI-H460 and its MX-selected derivative ABCG2-overexpressing cell line NCI-H460/MX20 and A549 cell line were grown as described previously [34]. The wild-type ABCG2-overexpressing cell line MCF-7/Flv1000 was maintained with 1,000 nmol/L of flavopiridol [35]. All cell lines were maintained in RPMI 1640 or DMEM medium (Hyclone Co., South Logan, Utah, USA), containing 10% fetal bovine serum and 1% penicillin/streptomycin and cultured at 37 °C in the incubator with 5% CO2.

### MTT cytotoxicity assay

Cytotoxicity tests and reversal experiments were performed using the MTT colorimetric assay as described previously [36]. Cells were harvested and resuspended in a final concentration of 6 × 10^3^ cells/well for the HEK293/pcDNA3.1, HEK/ABCB1, HEK/ABCC10, ABCG2-482-R2, ABCG2-482-T7 and ABCG2-482-G2 cells, and 4 × 10^3^ cells/well for the A549, NCI-H460 and NCI-H460/MX20 cells. Cells were evenly seeded into 96-well microtiter plates. After 24 h, various concentrations of Icotinib, pemetrexed and oxaliplatin were added into the each well. To evaluate the synergistic cytotoxic activity of Icotinib and pemetrexed combination, serial concentrations of Icotinib (0 ~ 30 μM) and pemetrexed (0 ~ 300 μM) were added simultaneously into each well. For the reversal experiments, various concentrations of chemotherapeutic drugs were added into designated wells after 2 h of preincubation with Icotinib, FTC, verapamil or cepharanthine. Then MTT solutions (4 mg/mL) were added after 68 h of incubation. The plate was incubated for an additional 4 h, and then the supernatant was discarded and 100 μl of DMSO were added to dissolve the formazan crystals. Cell viability was measured using an OPSYS microplate Reader from DYNEX Technologies, Inc. (Chantilly, VA) at 570 nm wavelength. All experiments were repeated at least 3 times, and the mean and standard deviation (SD) value were calculated.

### [^3^H]-MX accumulation and efflux assay

The effect of Icotinib on the intracellular accumulation and efflux of [^3^H]-MX was examined in ABCG2-overexpressing cells as previous described [37]. Briefly, the cells (5 × 10^6^/cells) were resuspended and incubated in the RPMI 1640 medium in the presence or absence of Icotinib (1.0 and 5.0 μM) or FTC (5.0 μM) at 37 °C for 2 h. Then cells were incubated with 0.01 μM [^3^H]-MX containing medium for an additional 2 h at 37 °C, with or without Icotinib (1.0 and 5.0 μM) or FTC (5.0 μM), and subsequently washed twice with ice-cold PBS. For accumulation assays, cells were lysed by the 10 mM lysis buffer (pH 7.4, containing 1% Triton X-100 and 0.2% SDS) and then placed in scintillation fluid. For the efflux assay, the suspended cells were continued to culture in the [^3^H]-MX free medium with or without Icotinib (5.0 μM) or FTC (5.0 μM) at 37 °C. The aliquots of cells were harvested at the indicated times (0, 30, 60, and 120 min), and then washed with ice-cold PBS and transferred to scintillation vials. The radioactivity was measured using the Packard TRI-CARB1 1900CA liquid scintillation analyzer from Packard Instrument Company, Inc (Downers Grove, IL). [^3^H]-MX (23 Ci/mmol) was a product of Moravek Biochemicals, Inc (Brea, CA).

### Western blot analysis

Western blotting was performed as previous described [38]. Monoclonal ABCG2 antibody (BXP-21) (sc-58222), DHFR antibody (E-18) (sc-14778) and the secondary horseradish peroxidase-labeled anti-mouse IgG were purchased from Santa Cruz Biotechnology, Inc. (Santa Cruz, CA). AKT (pan) (C67E7) Rabbit mAb #4691, phospho-AKT (Thr308) (C31E5E) Rabbit mAb #2965, E2F-1 Rabbit mAb #3742, Thymidylate synthase (D5B3) Rabbit mAb #9045, GAPDH (D16H11) Rabbit mAb #5174 and the secondary horseradish peroxidase-labeled anti-rabbit IgG were purchased from Cell Signaling Technology Inc (USA). Protein quantified expression was measured by ImageJ 1.47 Software (NIH, USA).

### Immunofluorescence

The immunofluorescence assay was carried out as described previously [39]. Cells (1 × 10^4^) were seeded into the 96-well plate and cultured with medium containing Icotinib (5.0 μM). After 72 h of incubation, cells were fixed with 4% paraformaldehyde (15 min) after washed with PBS, and then blocked with the BSA (2 mg/ml) for 1 h, followed by incubation with monoclonal antibody BXP-21 (against ABCG2) (1:200 dilution) overnight. Alexa flour 488-conjugated goat anti-mouse IgG (1:1000) (Molecular Probes, Carlsbad, CA) was used as the secondary antibody. Cell nuclei were counterstained by DAPI. Immunofluorescence images were caught by inverted microscope (model IX70; Olympus, Center Valley, PA) with IX-FLA fluorescence and CCD camera.

### ABCG2 ATPase assay

Crude membrane protein (100 μg protein/mL) from High-five cells expressing ABCG2 was incubated at 37°C with the indicated concentration of Icotinib in the presence and absence of beryllium fluoride (0.2 mmol/L beryllium sulfate and 2.5 mmol/L sodium fluoride) in ATPase assay buffer (50 mmol/L KCl, 5 mmol/L NaN_3_, 2 mmol/L EGTA, 10 mmol/L MgCl_2_, 1 mmol/L DTT, pH 6.8) for 10 min. The specific ATPase activity was recorded as beryllium fluoride–sensitive ATPase activity as described previously [40].

### Photoaffinity labeling of ABCG2 with [^125^I]-IAAP

Crude membranes (1 mg protein/mL) from ABCG2-expressing MCF-7 FLV1000 cells were incubated with 0 to 50 μmol/L of Icotinib for 10 min at 21°C to 23°C in 50 mmol/L Tris-HCl (pH 7.5). The photo-crosslinking of ABCG2 with 3 to 6 nmol/L [^125^I]-IAAP (2,200 Ci/mmol) followed by immunoprecipitation with BXP-21 was done as previously described [40]. Immunoprecipitated ABCG2 protein crosslinked with [^125^I]-IAAP was resolved on a 7% Tris-acetate gel. The incorporation of [^125^I]-IAAP into the ABCG2 band was quantified using the STORM 860 PhosphorImager system (Molecular Dynamics) and the software ImageQuaNT, as described [40].

### Molecular modeling

The structures of Icotinib and ABCG2 homology model were refined using our previous protocols [41]. Glide v6.0 (Schrödinger, LLC, New York, NY, 2013) was used with the default functions. All computations were performed on Dell Precision 490n with the Linux OS (Ubuntu 12.04 LTS).

### Animals

Athymic NCR (nu/nu) nude mice, 10 to 15 weeks old and weighing 18 to 22 g (Taconic Farms (NCRNU-M, Homozygous, Albino color)), were used for the ABCG2 xenograft models. All animals were provided with sterilized water and rodent chow ad libitum and maintained with an alternating 12 h light/dark cycle. All the experiments were approved by the Institutional Animal Care & Use Committee (IACUC) of St. John's University, and were carried out in accordance with the guidelines from Animal Welfare Act and The U.S. Public Health Service *Policy on Humane Care and Use of Laboratory Animals*.

### Nude mouse MDR xenograft models

The ABCG2-overexpressing NSCLC cell NCI-H460/MX20 xenograft models were established as previously described [41]. Briefly, NCI-H460/MX20 cells (4 × 10^6^) were injected subcutaneously under the armpits region of the nude mice. The mice were randomized into 6 groups (n = 8) when the tumors reached a mean diameter of 0.5 cm (day 0), and then received treatments as follows: (a) vehicle (saline + 0.5% Sodium Carboxymethyl Cellulose (CMC-Na)) (q3d × 6), (b) Icotinib diluted in saline + 0.5% CMC-Na (60 mg/kg, p.o., q3d × 6), (c) pemetrexed (100 mg/kg, i.p., q3d × 6), (d) Icotinib (60 mg/kg, p.o., q3d × 6, given 2 h before giving pemetrexed) + pemetrexed (100 mg/kg, i.p., q3d × 6), (e) topotecan (3.0 mg/kg, i.p., q3d × 6), and (f) Icotinib (60 mg/kg, p.o., q3d × 6, given 2 h before giving topotecan) + topotecan (3.0 mg/kg, i.p., q3d × 6). The body weights of the mice were monitored and the two perpendicular diameters of tumors (*A* and *B*) were recorded every 3rd day, the tumor volumes (*V*) were calculated according to the following formula described previously [41].

$$V=\frac{\pi }{6}{\left(\frac{A+B}{2}\right)}^{3}$$

The ratio of growth inhibition (IR) described previously [41] was estimated according to the formula given below.

$$\text{IR}(\%)\;=\;1-\frac{Mean\;tumor\;weight\;of\;\exp erimental\;group}{Mean\;tumor\;weight\;of\;control\;group}\;\times \;100\%$$

### Immunohistochemistry (IHC)

Tumors were collected after 18 days of treatment and Immunohistochemistry were performed as previously described [42]. The primary monoclonal antibody against ABCG2 (BXP-21) at the dilution of 1:100 and labeled polymer-HRP anti-mouse (DAKO) secondary antibody were used in the experiment. Nikon Eclipse E600 microscope with NIS Elements D3.0 software was used to catch the image.

### Statistics

Statistical analysis was calculated using the Student's t test. *P* < 0.05 was considered statistically significant.

## SUPPLEMENTARY TABLE


