# Supplementary material for: Icotinib antagonizes ABCG2-mediated multidrug resistance, but not the pemetrexed resistance mediated by thymidylate synthase and ABCG2
Source: Oncotarget. 2014 Jun 13;5(12):4529–42. doi: 10.18632/oncotarget.2102 (PMC4147343; doi:10.18632/oncotarget.2102)
Supplement: Supplementary file 1 [file oncotarget-05-4529-s001.pdf]

Icotinib antagonizes ABCG2-mediated multidrug resistance, but not the pemetrexed resistance mediated by thymidylate synthase and ABCG2

Supplementary Material

Supplementary Table S1: The reversal efficacy of Icotinib in ABCB1- and ABCC10-mediated drug resistance.

Compounds	$IC_{50} \pm SD^a$ (μM)					
	HEK293/pcDNA3.1	(RF)[b]	HEK/ABCB1	(RF)[b]	HEK/ABCC10	(RF)[b]
Vincristine (μM)	0.0128 ± 0.0006	1.0	0.1793 ± 0.0143	14.0	0.0896 ± 0.0072	7.0
+ Icotinib 1.0 μM	0.0118 ± 0.0008	0.9	0.1738 ± 0.0104	13.6	0.0827 ± 0.0074	6.5
+ Icotinib 5.0 μM	0.0125 ± 0.0010	1.0	0.1624 ± 0.0073	12.7	0.0812 ± 0.0041	6.3
+ Verapamil 5.0 μM	0.0129 ± 0.0006	1.0	0.0150 ± 0.0011*	1.2		
+ Cepharanthine 2.5 μM	0.0104 ± 0.0009	0.8			0.0166 ± 0.0007*	1.3

a. IC_{50} values are represented the mean ± standard deviation (SD).
b. Resistance fold (RF) was calculated by the IC_{50} values for vincristine of HEK293/pcDNA3.1 cell with Icotinib or known reversing agents, or the resistant cell lines in the presence or absence of Icotinib or reversing agents, divided by the IC_{50} values for vincristine of HEK293/pcDNA3.1 without the reversing agents.
*. $P < 0.05$, versus the control group.
